# Climate change on the Tibetan Plateau in response to shifting atmospheric circulation since the LGM

**DOI:** 10.1038/srep13318

**Published:** 2015-08-21

**Authors:** Liping Zhu, Xinmiao Lü, Junbo Wang, Ping Peng, Thomas Kasper, Gerhard Daut, Torsten Haberzettl, Peter Frenzel, Quan Li, Ruimin Yang, Antje Schwalb, Roland Mäusbacher

**Affiliations:** 1 Key Laboratory of Tibetan Environment Changes and Land Surface Processes (TEL), Institute of Tibetan Plateau Research (ITP), Chinese Academy of Sciences, Beijing, China; 2 CAS Center for Excellence in Tibetan Plateau Earth System, Beijing, China; 3 Institute of Geography, Friedrich-Schiller-University Jena, Germany; 4 Institute of Earth Sciences, Friedrich-Schiller-University Jena, Germany; 5 Institute of Geographical Sciences and Natural Resource Research, Chinese Academy of Sciences, Beijing, China; 6 Institute of Geosystems and Bioindication, Braunschweig University of Technology, Braunschweig, Germany

## Abstract

The Tibetan Plateau (TP) is primarily influenced by the northern hemispheric middle latitude Westerlies and the Indian summer monsoon (ISM). The extent, long-distance effects and potential long-term changes of these two atmospheric circulations are not yet fully understood. Here, we analyse modern airborne pollen in a transition zone of seasonally alternating dominance of the Westerlies and the ISM to develop a pollen discrimination index (PDI) that allows us to distinguish between the intensities of the two circulation systems. This index is applied to interpret a continuous lacustrine sedimentary record from Lake Nam Co covering the past 24 cal kyr BP to investigate long-term variations in the atmospheric circulation systems. Climatic variations on the central TP widely correspond to those of the North Atlantic (NA) realm, but are controlled through different mechanisms resulting from the changing climatic conditions since the Last Glacial Maximum (LGM). During the LGM, until 16.5 cal kyr BP, the TP was dominated by the Westerlies. After 16.5 cal kyr BP, the climatic conditions were mainly controlled by the ISM. From 11.6 to 9 cal kyr BP, the TP was exposed to enhanced solar radiation at the low latitudes, resulting in greater water availability.

Because of its large area and high elevation, the TP affects the mechanical and thermodynamic properties of its overlying air masses^1^. Therefore, this process influences climatic conditions over a wide area of Southern and Eastern Asia^2^,^3^. Over long time scales (tens of thousands of years), the intensities to which the Westerlies and ISM have affected the TP are assumed to have varied with changing global climatic backgrounds. These effects have resulted in different hydrological and energy budgets and different thermodynamic reactions^4^ of the TP. Changes that have occurred in the NA since the LGM may have influenced the TP in two ways: (1) Comparisons between palaeoclimatic conditions inferred from the Chinese Loess Plateau and from the NA (Greenland ice-cores) indicate that the climatic signal may have been directly transmitted to the TP through the zonal mid-latitude Westerlies^5^. (2) Climatic variations in the NA may have also affected the energy budget of the tropical Indian Ocean via thermohaline circulation (THC), thus reaching the TP through the enhancing and weakening of the ISM^6^. Although the results of previous paleo-climatic investigations on the TP^7^,^8^,^9^ often correspond to typical climatic events derived from marine sediments of the NA^10^ and Greenland ice cores^11^, no clear explanation has yet been proposed for the coupling mechanisms and processes between the TP and the NA regarding the climate signal transfer^12^. The primary problem is that only a few convincing proxies are available that can differentiate the influence of the Westerlies and/or the ISM^13^ on the climate of the TP. The central TP represents a transition zone that is dominated by the Westerlies during the winter and spring and by the ISM during the summer and fall^14^. Prior to reaching the TP, the Westerlies have to cross large portions of Eurasia, whereas air masses related to the ISM reach the TP from the south after crossing only small areas of the Indian subcontinent. These two pathways vary significantly not only in their general source area (Westerlies, northern hemisphere mid-latitudes; ISM, tropical Indian Ocean) but also in their distance to water sources (oceans). Thus, both atmospheric circulation systems differ in the moisture they transport. Although the Westerlies reaching the TP are characterized by low moisture, the ISM brings substantial precipitation to this generally semi-arid region. Because the vegetation assemblages in the course of these pathways are different, the airborne pollen collected on the central TP has great potential as an indicator to trace these different pathways. Therefore, modern airborne pollen was collected for one year (Sept. 2005 to Aug. 2006) at Lake Nam Co (a large lake on the central TP) and used to establish a pollen discriminant index (PDI) to distinguish between the influences of the Westerlies and ISM. This PDI is interpreted in terms of the wind direction and thus acts as a proxy for the dominating atmospheric circulation system. The results of the modern PDI were subsequently applied to the pollen spectra gained from a continuous lacustrine sediment record collected from Lake Nam Co to interpret shifts in the atmospheric patterns since the LGM. Furthermore, using the PDI in combination with proxies for biological productivity and hydrological variation, the environmental response to climatic change caused by the varying Westerly and ISM influence can be interpreted.

The lacustrine core (NC 08/01, 10.41 m length) was recovered from the centre of Lake Nam Co at a water depth of 93 m (Fig. 1). Chronological control is based upon 24 AMS radiocarbon ages in combination with radio isotopic (^210^Pb, ^137^Cs) measurements and magnetostratigraphy for the upper portion. Modern airborne pollen was collected over the period from September 1^st^, 2005 to August 31^st^, 2006. Pollen concentrations and pollen types within each sample were identified^15^, and their relationships plus local wind direction and frequency, near surface wind field and backward air mass trajectories were used to establish the PDI. To confirm the consistency between the airborne pollen assemblages from the traps and those deposited in the lake sediments, the annual airborne samples were compared with simultaneously obtained lake surface sediment samples. Applying identical palynological methods to the 24 cal kyr BP sedimentary record from Lake Nam Co resulted in a Westerly-ISM discrimination index. Total organic carbon (TOC), total nitrogen (TN) and their molar ratio (C/N) were used to interpret the source of the organic matter and its environmental significance. The elemental concentrations of *Ca* and the ratio of *Fe* and *Mn* are used to indicate hydrological (lake level) changes to support the pollen-derived proxies.

## Results

Three independent methods, i.e., radiocarbon dating, radio isotopic measurements and magnetostratigraphical support, were used to establish a detailed chronology (Supplementary, Tab. S1). Based on the age-depth model (Supplementary, Fig. S1), the core covers the past 24 cal kyr BP with highly variable sediment accumulation rates^16^,^17^.

Records of the Nam Co weather station indicate that the prevailing wind direction is WWS (ca.255°) during the spring and winter, SSW (ca.195°) in the autumn, and south (ca.180°) in the summer^18^ (Supplementary, Fig. S2). The distribution of airborne pollen grains is generally influenced by the phenophase of the local plants^15^ and by these prevailing wind directions^19^. Hence, the pollen assemblages vary significantly with the season of deposition (Fig. 2). Because *Pinus* and *Picea* do not presently grow in the Nam Co catchment or its adjacent areas, these airborne pollen taxa are considered to be exotic (long distance). During the summer and fall, *Pinus* and *Picea* pollen may originate from the south or south-east, but these taxa occupy only a small proportion of the pollen assemblages because dominant local herbaceous taxa bloom during this period. However, in the winter, with the wind direction changing from south-southeast to west, and in the spring, with the prevailing western wind, the proportion of *Pinus* and *Picea* in the pollen assemblages is remarkably greater^15^. Therefore, the prevailing wind direction is assumed to be the primary reason for the presence of these exotic pollen taxa in the winter.

To clarify the relationship between the prevailing wind directions and the atmospheric circulation, we reconstruct the near surface wind field using the reanalysis data and methods of the NCEP/NCAR (US National Centers for Environmental Prediction/National Center for Atmospheric Research)^20^; we also recreate the backward air mass trajectories during the identical period by applying the HYSPLIT 4 Model^21^ to indicatethe relevant atmospheric circulation statuses. The reconstructed near surface wind fields and the main air masses display different features during different seasons. The near surface wind fields indicate the atmospheric circulation directions. These atmospheric circulation directions and air masses primarily originate from the north and west in the spring, and the source areas may reach the Altai Mountains and Central Asia. In winter, these air masses originate from the western sector and can be tracked to Southern Europe. In summer, the winds are primarily sourced to the Bay of Bengal and, on the continent, south of the Himalayas. During autumn, the prevailing wind direction is south and southwest but with short transportation distances (Supplementary, Fig. S3, Fig. S4). Generally, seasonal distributions of atmospheric circulation directions and backward air mass trajectories are identical to the prevailing wind directions in the Nam Co area. Thus, airborne pollen in the Nam Co area can be interpreted as being dominated by atmospheric circulation directions. Air masses and hence the airborne pollen assemblage is driven by the zonal Westerlies in the spring, winter and a portion of autumn, whereas in the summer and the remaining portion of autumn, the ISM is the main delivery source of airborne pollen grains.

To investigate the quantitative relationships between pollen assemblages and the dominant extent of different atmospheric circulations, we conducted discriminant analyses^22^ to classify 19 major airborne pollen taxa (representing 70.68% of all airborne taxa) that also appeared in the NC08/01 core.

The results of the discriminant analysis, which are divided into two groups (A and B), indicate that the centroid of the airborne pollen assemblages of group A from the ISM period (May–Sep.) is −0.8931 (negative), whereas that of group B from the Westerlies period (Oct.–Apr.) is 0.7095 (positive). We use these scores as a pollen discriminant index (PDI), where lower values represent an enhanced ISM influence and higher values indicate stronger Westerlies (Fig. 2).

To understand the relationships between airborne pollen and modern pollen assemblages stored in the sediments in the lake, we collected ten lake surface sediment samples, with uniform spatial distribution but from different depths in the lake, and identified their pollen taxa. The pollen assemblages in the surface sediments correlate well with the annual airborne pollen samples (Supplementary, Fig. S5, Tab. S2). Because the climatic conditions recorded for the sampling period between 2005 and 2006 are in the normal range of the long-term period between 1971 and 2010^23^, the annual airborne pollen distribution can generally be regarded as representative of several decades. Thus, the well-preserved pollen assemblages collected from the 24 cal kyr BP sediment record from Lake Nam Co and the PDI derived from their analysis are excellent recorders of climatic and environmental signals driven by the dominant atmospheric circulation pattern.

## Discussion

Environmental, climatic and circulation proxies derived from the sedimentary record of Lake Nam Co reveal high variability throughout the examined period (Fig. 3). However, the most prominent shift in the palynological proxies, likely indicating a shift in the atmospheric circulation system, occurs at ca. 16.5 cal kyr BP (Fig. 3). Between 24 and 16.5 cal kyr BP (I-1 and I-2, Fig. 3), a decrease in humidity-preferring Cyperaceae and an increase in arid-preferring *Artemisia*^24^ indicate shrinkage in the wetland areas around the lake. A continuous decrease in *Pediastrum* indicates fewer nutrients in the lake water^25^, possibly caused by the inflow of nutrient-poor glacial melt water^26^ into Lake Nam Co. The volume and thus the surface area of Lake Nam Co are assumed to have increased, as suggested by the previously observed decreases in sediment accumulation rates after 19.5 cal kyr BP^17^ (Fig. S1). Although the total pollen concentration (TPC) is lower, *Pinus* and *Picea* account for greater than 30% of the pollen. Because the pollen of these species is regarded as exotic and is highly correlated with a positive PDI, this high abundance indicates that the lake catchment has been dominantly influenced by the Westerlies (Fig. 4a). The Westerlies even appear to increase in strength approximately 20.5 cal kyr BP, likely resulting from an advance of the polar ice sheet during the LGM^5^.

During the LGM, the dipole-shaped sea surface temperature (SST) and rainfall anomaly in the Atlantic region^27^ created warmer and moister conditions in the southern Atlantic Ocean. These anomalies in the southward shift of the intertropical convergence zone (ITCZ) excite a wave-train perturbation across Africa and induce warmer and moister conditions in the southern Indian Ocean^28^. This shift weakens the southwest wind of the ocean and enhances upwelling in the west Arabian Sea, which also leads to a higher TOC concentration in the SO90-111KL core from the Arabian Sea of this area (Fig. 4c)^29^. This process likely enables more moisture to be transported to the Arabian Peninsula and Iran Plateau, benefiting vegetation development. Therefore, the subtropical westerly jet may transport more exotic pollen to the TP and result in an increased exotic pollen ratio in the Nam Co catchment. During the northern hemisphere deglaciation from 20.5 to 16.5 cal kyr BP (I-2, Fig. 3), climatic conditions also favour vegetation growth within the catchment of Lake Nam Co, resulting in greater proportions of local species and hence fewer exotic pollen types being deposited in the lake. The PDI values imply that the influence of the ISM was gradually enhanced in the Lake Nam Co area during this period.

From 16.5 to 14.2 cal kyr BP (II-1, Fig. 3), the increase in the moisture-preferring Cyperaceae and Gramineae and the decrease in the arid-preferring *Artemisia* and Chenopodiaceae indicate that the climate in the lake catchment became more humid^30^. Low C/N ratios during this period imply an endogenic source for the TOC^31^, and the low TOC values indicate a low total biogenic productivity because the cold climatic conditions are unfavourable for terrestrial vegetation^32^. However, the proportion of exotic pollen (*Pinus* and *Picea*) drops, and the more negative PDI values indicate that the ISM is already dominant in the Lake Nam Co region. Studies from the NA focus on the Atlantic Meridional Overturning Circulation (AMOC) slowdown (Fig. 4d,e)^6^ or shutdown (Fig. 4f)^33^ during this period, which is assumed to correspond to Heinrich event 1 (H1). The cooling in the NA might have affected the Westerlies in strength and position. This Westerly-strengthening signal might have reached the TP via the pathway of the air masses crossing large portions of the Eurasian continent. The consequently dry Westerlies could have only minimally increased the humidity on the TP, and thus the Nam Co area was affected by cool and dry conditions. During the H1 stage, the dipole-shaped SST and rainfall anomaly also produced warmer and moister conditions in the southern Atlantic Ocean. This anomaly exerts an influence on the Indian Ocean via thermohaline circulation, which also leads to warmer and moister conditions in the southern Indian Ocean and the dry climate of the northern Indian Ocean. This effect is apparent in the increases in SST and precipitation recorded in the cores of the tropical Indian Ocean (Fig. 4d,e)^6^; only SST increased in the Bay of Bengal (Fig. 4g)^34^. These dry air masses may have forced the ISM to shift northward, bringing limited moisture to the TP. The decreased moisture in this period is also supported by the heavy oxygen isotope values of stalagmites in the Dongge Cave (Fig. 4h) in southwest China^35^.

Between 14.2 and 13.2 cal kyr BP (II-2, Fig. 3), in addition to the continuous increase in moisture-preferring Cyperaceae, the rising TPC and TOC values (Fig. 3) indicate an increase in vegetation biomass in the lake catchment. The increasing *Fe*/*Mn* ratio indicates anoxic conditions during sediment formation and/or even within the lake^36^. The decrease in *Ca* seems to result from more fresh water input and hence less carbonate precipitation. Both effects are apparently geochemical responses to a lake level rise^37^. This time span corresponds to the Bølling-Allerød (B/A) interstadial, which is also conspicuous in Lake Nam Co. A simulation study provided strong evidence that the B/A interstadial can be attributed to enhanced AMOC^38^. In this period, similar conditions in the southern and northern Atlantic Ocean lead to an increase in water temperature in the northern Indian Ocean upwelling current through thermohaline circulation, enhancing the ISM^39^.

Between 13.2 and 11.5 cal kyr BP (II-3, Fig. 3), the TPC and Cyperaceae decrease as the Chenopodiaceae increase, implying that vegetation development in the lake catchment was limited by reduced water availability. The lower *Fe*/*Mn* ratio and an increase in *Ca* because of carbonate precipitation could reflect this change in the precipitation/evaporation (P/E) balance and suggest a possible decrease in the lake level. The peak in TOC at ca. 12.5 cal kyr BP can be attributed to enhanced input of terrestrial organic material into the lake as the maximum C/N ratios occur contemporaneously. The subsequent decrease in TOC implies a cold climate in this time. All parameters indicate a colder and drier period corresponding to a widely reported Younger Dryas (YD) event^17^. However, PDI values suggest that the lake area is still dominated by the ISM. No evidence exists of an AMOC shutdown since the H1 stage, even during the YD period^32^. Although the AMOC might have weakened during the YD period, ISM was still dominant in the North Indian Ocean, Indian sub-continent and even further north because of the dipole-shaped SST and the rainfall anomaly in the Atlantic Ocean and because of the limited temperature decrease in the North Indian Ocean upwelling through thermohaline circulation^39^.

Both TPC and TOC undergo a rapid increase and reach their maxima between 10.2 and 9.3 cal kyr BP (III-1, Fig. 3), whereas xeric Chenopodiaceae sharply drop in abundance and stabilize at lower values, indicating a warm, moist stage that supports terrestrial vegetation and lake plankton development. The highest *Fe*/*Mn* ratio and the lowest *Ca* values suggest that the lake level had apparently increased quickly to its maximum during this period (Fig. 3). Two dry periods are reflected by increased *Ca* contents: from 8.0 to 6.0 cal kyr BP in the middle Holocene and from 2.0 to 1.3 cal kyr BP in the late Holocene (Fig. 3). During the former period, a slight decrease in TPC can be attributed to dry conditions that limit vegetation development. During the latter period, the decrease in both TPC and TOC implies a cold, dry environment that decreases the lake area. Within the Holocene, the climate on the central TP (Fig. 4b) is different from that of the Northern Hemisphere as reflected by the NGRIP Greenland ice core (Fig. 4i). The thermohaline circulation does not change the north Indian Ocean upwelling because of the stable AMOC. Greater solar radiation in the low latitude zone (Fig. 4j)^40^ increases the SST of the tropical Indian Ocean, potentially resulting in an enhanced ISM and warmer and moister conditions on the TP than in the NA.

Our results indicate that these environmental changes and climatic events on the TP are highly coincident with those of the NA since the LGM. However, these factors have different connecting mechanisms under different controlling atmospheric circulation systems. From 24 to 16.5 cal kyr BP, the TP is dominated by an enhanced southward shift of the zonal Westerlies, through which climatic features from the NA may have been transmitted to the TP. Between 16.5 and 11.5 cal kyr BP, the Westerlies might have transmitted only temperature signals to the TP, whereas the precipitation on the TP, which controls vegetation development, likely was driven by the ISM. In the beginning of the Holocene, an increase in solar radiation in the low latitude zone may have enhanced the ISM, inducing the most positive P/E balance on the TP during the entire studied period.

## Materials and Methods

### Study site

**Figure 1:**
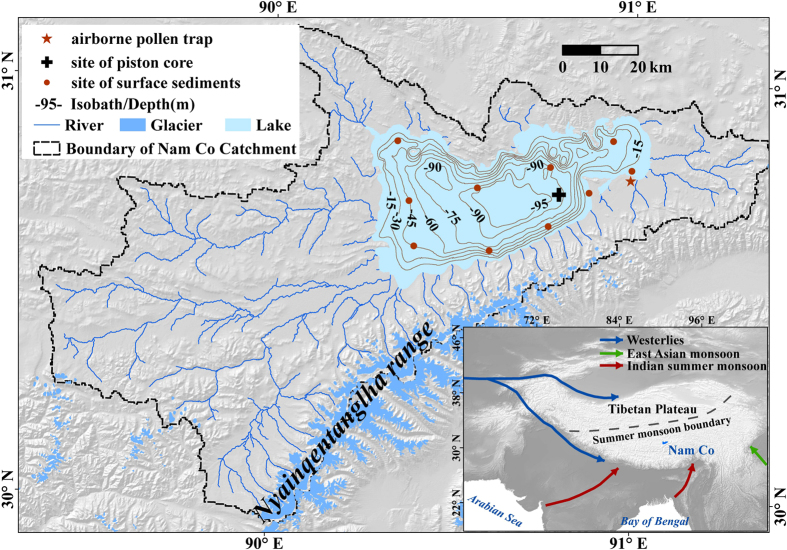
Map of the position of Lake Nam Co (including sites of the airborne pollen trap, surface sediment samples and the investigated core, NC 08/01). The inset shows the main atmospheric circulation systems influencing the TP. The terrain map of the catchment was generated using ArcGIS 10.0 based on the Shuttle Radar Topography Mission Digital Elevation Model (SRTM DEM) from the U.S. Geological Survey. (https://lta.cr.usgs.gov/citation). The isobath lines of the lake were generated based on 305720 *in situ* survey points of the water depth (performed by the authors).

**Figure 2:**
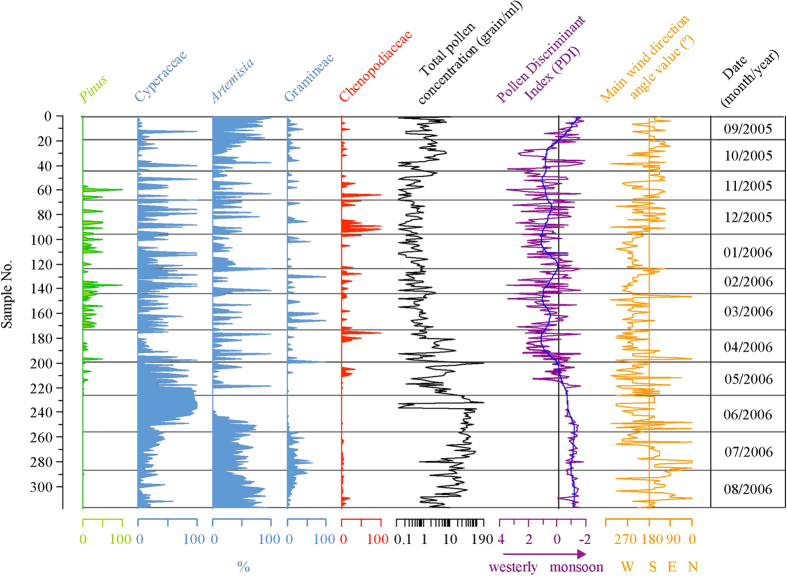
The major airborne pollen taxa (relative abundances in %), the calculated PDI and the prevailing wind directions during the sampling period in the Nam Co catchment. The red line is the 5 point smoothed PDI, with negative values indicating enhanced ISM and positive values indicating enhanced Westerlies. The grey horizontal lines are the group centroids based on the respective discriminant scores. The main wind directions are expressed as the angle of circumference (0° = North, 90° = East, 270° = South and 360° = West.

**Figure 3:**
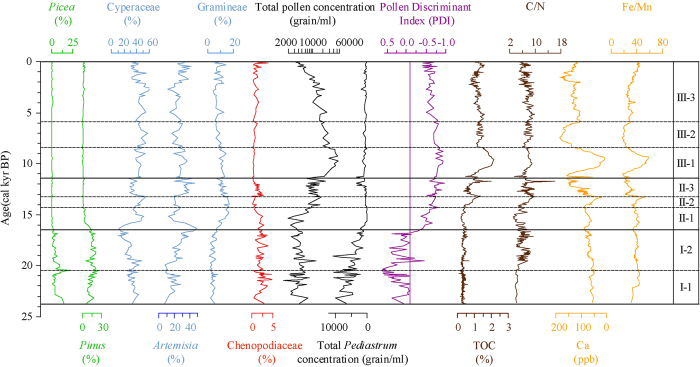
Comparisons of environmental proxies from Lake Nam Co during the past 24 cal kyr BP. The PDI exhibits a clear shift at 16.5 cal kyr BP, revealing a change in domination from the Westerlies to the ISM. TOC is a proxy for biological productivity, and C/N indicates the origin of the organic matter (higher values = terrestrial, lower values = aquatic), *Ca* is a proxy for lake level variations (high values = low lake level, low values = high lake level), and *Fe*/*Mn* is an indicator of redox conditions, which are coupled to the lake level stage.

**Figure 4:**
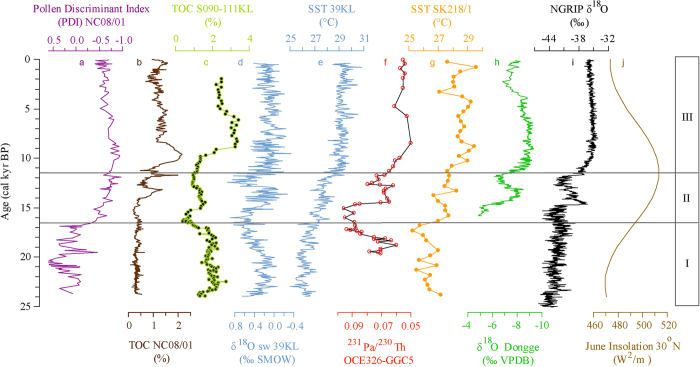
Comparisons of the PDI (**a**) and TOC (**b**) from Lake Nam Co with other records from the North Atlantic (NA) and the Indian Ocean during the past 24 cal kyr BP. (**c**) TOC of marine core SO90-111KL from the Arabian Sea^29^; (**d**,**e**), seawater δ^18^O and SST, respectively, of marine core 39KL from the eastern tropical Indian Ocean^6^. (**f**) ^231^Pa/^230^Th in the marine sediment core OCE326-GGC5 from the subtropical North Atlantic Ocean^33^. (**g**) reconstructed SST of marine core SK218/1 core from the western Bay of Bengal^34^. (**h**) δ^18^O of a stalagmite from the Dongge Cave located in southwest China^35^. (**i**) δ^18^O of Greenland ice core NGRIP^11^. (**j**) June solar insolation pattern from 30°N^40^. The record locations are shown in Fig. S6.

Lake Nam Co, from which sediment core NC 08/01 was recovered using an Uwitec piston corer (http://www.uwitec.at), is situated at 90°16′-91°03′E and 30°30′-55′N. The present lake level is 4718 m asl, the surface area is 2,015 km^2^, and the catchment comprises 10,610 km^2^. The Nyainqentanglha Mountains, with an average elevation of approximately 5,500 m asl, are located at the south-eastern margin of the catchment. Consequently, precipitation reaches this area only during the late spring and summer (MJJAS), delivered primarily by the ISM^14^. The northern and north-western regions of the catchment are inland low relief mountains and hills with an average elevation of 5,000 m asl. This morphologic configuration allows the dry Westerlies to migrate easily into the Nam Co catchment during winter. Many current glaciers are distributed over the Nyainqentanglha Mountains (Fig. 1). Precipitation, land surface runoff, melt water and ground water contribute ca. 27%, 35%, 10% and 28%, respectively, of the water supply to the lake^41^. **Airborne pollens and PDI**: Modern airborne pollen grains were sampled by a Burkard pollen trap stationed 3 m above the ground on the south-eastern lake shore^15^. This trap is a volumetric air-suction device capable of continuously gathering airborne pollen and fungal spore particles. Air is drawn in at 10 litres/min, and airborne particles are deposited on a sticky tape mounted on a drum that completely rotates once a week. The tape on this rotating drum was renewed weekly and sampled for daily resolution by cutting the tape into seven pieces, with each piece representing one day of sampling. The PDI determination followed these steps: 1) 19 major taxa of airborne pollen (70.68% of all airborne taxa; also appeared in the NC08/01 core) are selected to perform discriminant analyses. These pollen types are *Picea*, *Pinus*, *Tsuga*, *Alnus*, *Betula*, *Artemisia*, Caryophyllaceae, Chenopodiaceae, Compositae, Cruciferae, Cyperaceae, Gentianaceae, Gramineae, Leguminosae, Labiatae, *Polygonum*, Ranunculaceae, Rosaceae, and *Thalictrum*. 2) Airborne pollen data for the entire year (a total of 323 samples because no pollen was collected on select days) are divided into two groups (A and B) according to the prevailing time of the atmospheric circulation: group A has 143 samples that represent the ISM period (May–Sep.) and group B has 180 samples that represent the Westerlies period (Oct.–Apr.). 3) Using discriminant analyses in SPSS (Statistical Package for the Social Sciences) 10.0, all 323 samples were classified into the predicted groups and cross-validated. The results indicate that 80.2% of samples were correctly classified in the predicated grouping and that 78.6% of samples were correctly classified in the cross-validated grouping (Supplementary, Tab. S3). 4) Using the discriminant function, we calculated the discriminant index of each sample and the centroids of each group, which represent the dominant atmospheric circulation status in that period. **Sampling and analyses of the core**: Based on the lithology and obtained sediment accumulation rates, pollen samples were selected in 5 to 30 cm intervals. Pollen grains were examined and identified using an Olympus light microscope at 400× magnification. The TOC, TN and element concentrations were measured at a 1 cm interval. The TOC and TN contents were measured using a Vario EL II (Elementar Analysensysteme GmbH), whereas *Fe*, *Mn* and *Ca* were measured using an ICP-OES (Varian).

## Additional Information

**How to cite this article**: Zhu, L. *et al.* Climate change on the Tibetan Plateau in response to shifting atmospheric circulation since the LGM. *Sci. Rep.* **5**, 13318; doi: 10.1038/srep13318 (2015).

## Supplementary Material

Supplementary Information
