# Supplementary material for: Climate change on the Tibetan Plateau in response to shifting atmospheric circulation since the LGM
Source: Sci Rep. 2015 Aug 21;5:13318. doi: 10.1038/srep13318 (PMC4543934; doi:10.1038/srep13318)
Supplement: Supplementary Information [file srep13318-s1.pdf]

Title:

Climate change on the Tibetan Plateau in response to shifting atmospheric circulation since the LGM

Authors:

Liping Zhu[1,2], Xinmiao Lü[1,2], Junbo Wang[1,2], Ping Peng[1], Thomas Kasper[3], Gerhard Daut[3], Torsten Haberzettl[3], Peter Frenzel[4], Quan Li[5], Ruimin Yang[1], Antje Schwalb[6], Roland Mäusbacher[3]

Author affiliations:

[1] Key Laboratory of Tibetan Environment Changes and Land Surface Processes (TEL), Institute of Tibetan Plateau Research (ITP), Chinese Academy of Sciences, Beijing, China

[2] CAS Center for Excellence in Tibetan Plateau Earth System, Beijing, China

[3] Institute of Geography, Friedrich-Schiller-University Jena, Germany

[4]Institute of Earth Sciences, Friedrich-Schiller-University Jena, Germany

[5]Institute of Geographical Sciences and Natural Resource Research, Chinese Academy of Sciences, Beijing, China

[6]Institute of Geosystems and Bioindication, Braunschweig University of Technology, Braunschweig, Germany

Corresponding author:

Liping Zhu, Key Laboratory of Tibetan Environment Changes and Land Surface Processes (TEL), Institute of Tibetan Plateau Research (ITP), Chinese Academy of Sciences, Beijing 100101, China, Tel: +86 10 84097093, E-mail: lpzhu@itpcas.ac.cn

Supplementary information

Tab. S1 AMS radiocarbon ages and reservoir effect corrected calibration results

Lab. No. (Beta -)	Sample-ID	Composite Depth (m)	Measured Radiocarbon Age (yr BP)	Conventional Radiocarbon Age (yr BP)	Reservoir Corrected Conventional Radiocarbon Age (yr BP)	2σ calibration (OxCal 4.1.7)		$^{13}C/^{12}C$ Ratio
						Median age (cal yr BP)	Error (±2σ)	
260883	NC08/01 Pilot 3 0-1 cm	0.005	1400±40	1420±40	0±40	-8	138	-23.8
269541	NC08/01 Pilot 3 35-36 cm	0.355	1920±40	1930±40	510±40	534	71	-24.1
277418	NC08/01 Pilot 3 46-47 cm	0.465	2480±40	2490±40	1070±40	975	64	-24.3
269542	NC08/01 Pilot 3 65-66 cm	0.655	2900±40	2910±40	1490±40	1372	106	-24.3
260872	NC08/01 [0-2] 0-1/60-61 cm	1.045	3530±40	3550±40	2130±40	2121	154	-23.6
269537	NC08/01 [0-2] 0-1/90-91 cm	1.345	4540±40	4540±40	3120±40	3348	96	-25.3
269536	NC08/01 [1-3] 1-2/30-31 cm	1.766	6680±40	6660±40	5240±40	5992	132	-26.3
260873	NC08/01 [1-3] 1-2/60-61 cm	2.066	9100±50	9060±50	7640±50	8437	84	-27.2
269538	NC08/01 [1-3] 1-2/90-91 cm	2.366	11320±60	11320±60	9900±60	11299	198	-25.3
271057	NC08/01 [2-4] 2-3/30-31 cm	2.782	12020±60	12060±60	10640±60	12589	137	-22.8
260874	NC08/01 [2-4] 2-3/60-61 cm	3.082	12800±70	12820±70	11400±70	13291	149	-23.5
271058	NC08/01 [3-5] 3-4/30-31 cm	3.121	13500±70	13530±70	12110±70	13937	177	-23.2
260875	NC08/01 [3-5] 3-4/60-61 cm	3.421	14860±80	14940±80	13520±80	16611	456	-20
291390	NC08/01 [3-5] 3-4/90-91 cm	3.721	15690±70	15750±70	14330±70	17049	266	-21.1
276418	NC08/01 [3-5] 4-5/0-1 cm	3.753	15130±70	15210±70	13790±70	17069	270	-20.4
277419	NC08/01 [3-5] 4-5/8-9 cm B	3.833	15470±70	15540±70	14120±70	17204	245	-20.9
276419	NC08/01 [3-5] 4-5/30-31 cm	4.053	16270±70	16340±70	14920±70	18178	315	-20.7
260876	NC08/01 [3-5] 4-5/60-61 cm	4.353	17440±90	17500±90	16080±90	19047	260	-21.1
260877	NC08/01 [5-7] 5-6/60-61 cm	5.274	18760±100	18850±100	17430±100	20323	289	-19.7
260878	NC08/01 [5-7] 6-7/60-61 cm	6.210	18570±100	18650±100	17230±100	20730	275	-20
260879	NC08/01 [7-9] 7-8/60-61 cm	7.179	18790±100	18870±100	17450±100	21108	253	-20.3
260880	NC08/01 [7-9] 8-9/60-61 cm	8.114	19380±100	19450±100	18030±100	21551	258	-20.5
260881	NC08/01 [9-11] 9-10/60-61 cm	9.089	19730±120	19800±120	18380±120	22174	248	-20.5
260882	NC08/01 [9-11] 10-11/60-61 cm	10.058	20280±120	20350±120	18930±120	23175	325	-21

Note: Twenty-four bulk samples were collected from the composite record NC 08/01 (containing the piston core NC 08/01 and a gravity core Pilot-3) for AMS radiocarbon dating and to obtain conventional radiocarbon ages. Another gravity core recovered from the identical position (Pilot-5) was used for the ^{137}Cs and ^{210}Pb measurements. A difference of 1420±40 yr in the top layer (0-1 cm) between the radiocarbon age of the bulk sample and the absolute age inferred by the sedimentary rate is considered to be a reservoir effect of the total NC 08/01 core. This reservoir effect is subtracted from all other data before radiocarbon ages are calibrated (2σ) using calendar ages in Calib® (Version 6.0) with the INTCAL 09 dataset (http://calib.qub.ac.uk/calib/) (Reimer *et al.* 2009). The reservoir was positively tested for the past 4,000 yr BP using magnetostratigraphy.

Reference:

Reimer, P. J. et al. IntCal09 and Marine09 radiocarbon age calibration curves, 0-50,000 years cal BP. Radiocarbon 51, 1111-1150 (2009).

Tab. S2 Correlation coefficients for the major pollen assemblages from the surface sediment samples and annual airborne pollen samples

Sample No.	NMSS07-12	NMSS07-16	NMSS07-17	NMSS07-21	NMSS07-25	NMSS08-1	NMSS08-4	NMSS08-8	NMSS08-16	NMSS08-41
NMSS07-16	0.974**	1								
NMSS07-17	0.986**	0.997**	1							
NMSS07-21	0.974**	0.999**	0.997**	1						
NMSS07-25	0.891**	0.828**	0.843**	0.825**	1					
NMSS08-1	0.939**	0.860**	0.882**	0.858**	0.980**	1				
NMSS08-4	0.819**	0.721**	0.744**	0.717**	0.982**	0.964**	1			
NMSS08-8	0.906**	0.832**	0.851**	0.829**	0.995**	0.992**	0.983**	1		
NMSS08-16	0.675**	0.659**	0.657**	0.655**	0.903**	0.809**	0.883**	0.871**	1	
NMSS08-41	0.874**	0.836**	0.843**	0.832**	0.987**	0.955**	0.958**	0.978**	0.935**	1
Annual airborne pollen	0.997**	0.962**	0.976**	0.961**	0.871**	0.932**	0.804**	0.892**	0.627**	0.847**

** Correlation is significant at the 0.01 level (2-tailed). * Correlation is significant at the 0.05 level (2-tailed).

Tab. S3 Summary statistics of the discriminant analysis results for the airborne pollen assemblages in the Lake Nam Co catchment

Actual group	Number of samples	Classified result				Cross-validation result			
		Predicted group	Number of samples	Correct rate	Average correct rate	Cross-validated group	Number of samples	Correct rate	Average Correct rate
A	143	A	132	92.3%	80.2%	A	129	90.2%	78.6%
		B	11			B	14		
B	180	A	53			A	55		
		B	127	70.6%		B	125	69.4%	

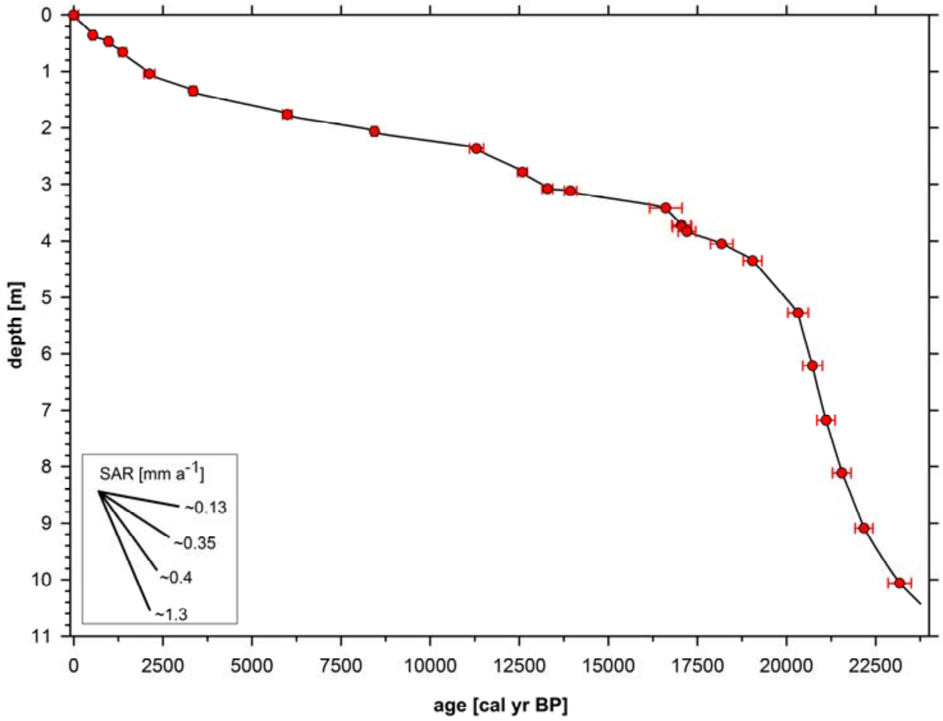

Fig. S1 Age-depth model of core NC 08/01. Red circles with error bars are reservoir-corrected calibrated median radiocarbon ages, and the solid line shows the interpolation between the single dates using the model output of OxCal 4.1.7. Based on the different slopes in the age-depth model, sediment accumulation rates (SAR) sharply decreased from approximately 1.3 mm/a to 0.38 mm/a at approximately 20.5 cal kyr BP and continuously decreased to only approximately 0.1 mm/a at approximately 16.5 cal kyr BP. The minimum phase lasted until approximately 11.6 cal kyr BP, indicating the beginning of the Holocene, which is characterized by a slight increase in SAR to approximately 0.36 mm/a.

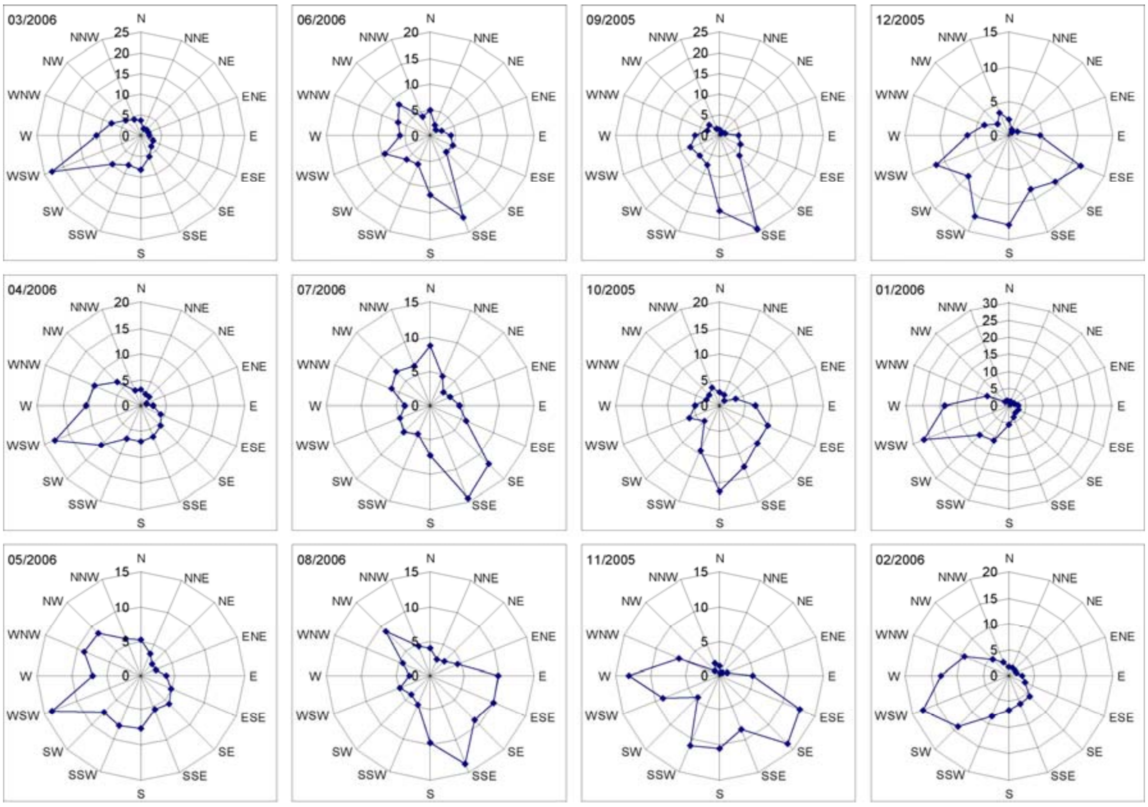

Fig. S2 Frequency of the wind direction in different seasons during the period between September 2005 and August 2006, collected from the weather station situated on the southern shore of Lake Nam Co. Columns 1-4 represent spring, summer, autumn and winter, respectively.

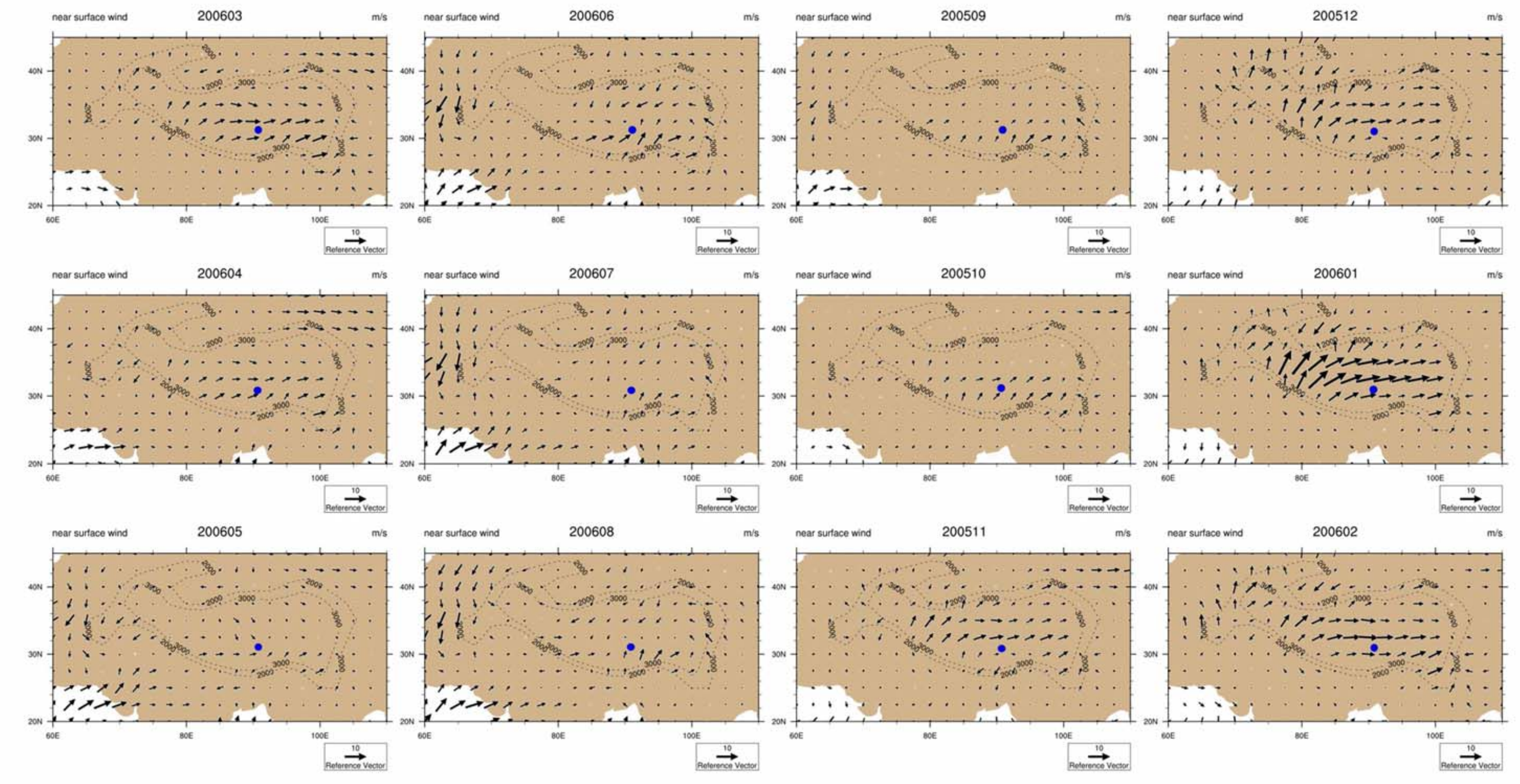

Fig. S3 Near surface wind field on the Tibetan Plateau from September 2005 to August 2006. Columns 1-4 represent spring, summer, autumn and winter, respectively. The lengths and directions of the arrows indicate the wind intensities and directions. The wind speed is in m/s. The near surface wind field is synthesized by monthly mean U and V components of the vertical layer with sigma = 0.995 (sigma = 1 is ground). The spatial resolution is 2.5° ×2.5°. The dotted lines are contour lines. The blue dot represents Lake Nam Co. The data originated from the NCEP/NCAR Reanalysis materials (www.esrl.noaa.gov/psd/data/gridded/data.ncep.reanalysis.html).

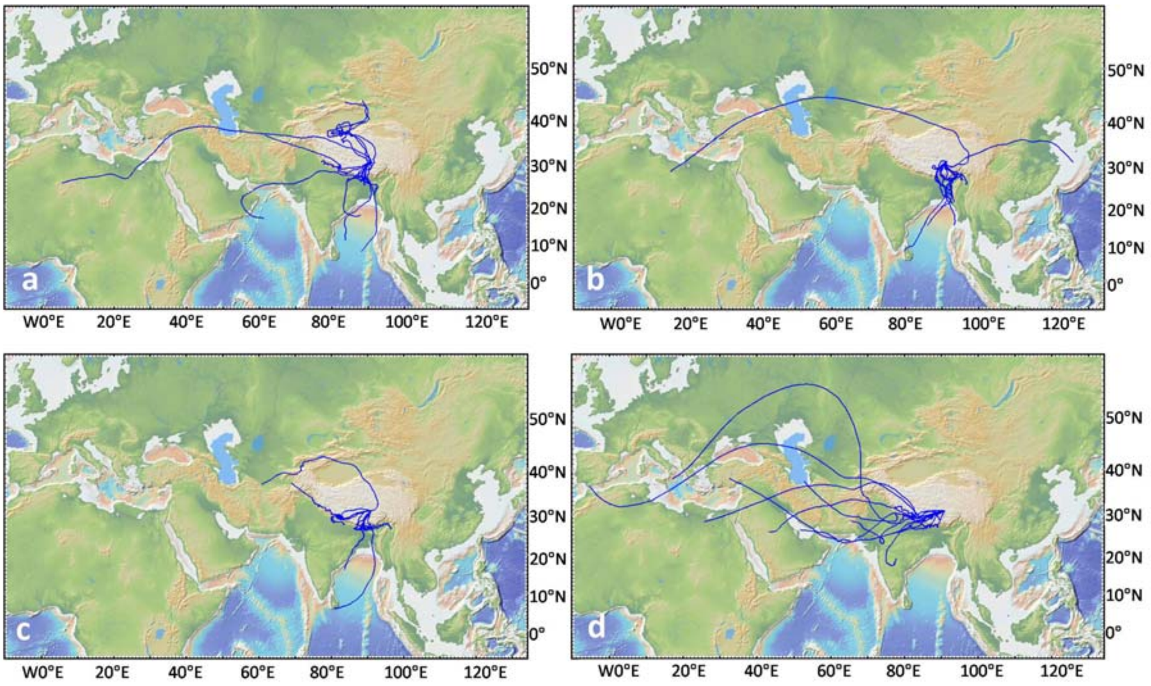

Fig. S4 Back trajectories of different seasons modelled for the Lake Nam Co area using HYSPLIT (http://ready.arl.noaa.gov/HYSPLIT.php). Meteorological data to run the model originate from the NCEP/NCAR global data assimilation system (GDAS). Backward air mass trajectories are calculated at timescales of 7 days at heights of 500 m above ground level (back trajectories were run every 6 hours). The base maps are generated with the GeoMapApp free software (http://www.geomapapp.org). An error of up to 20% is noted in the simulated trajectory distances. a. spring, b. summer, c. autumn, and d. winter.

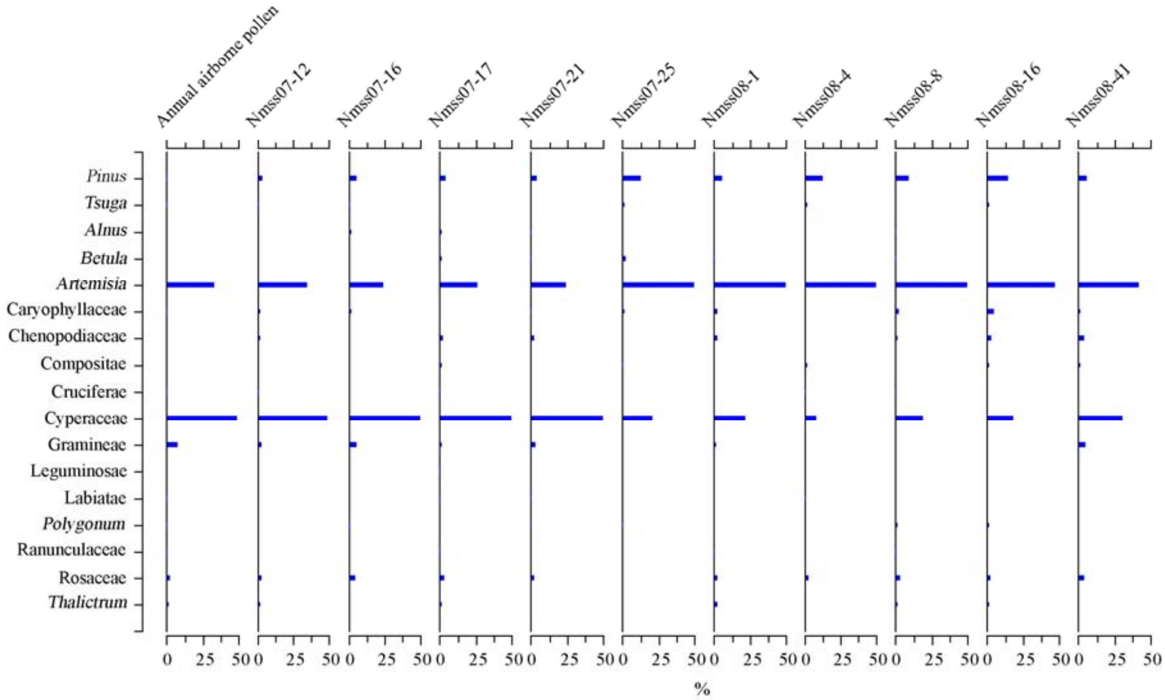

Fig. S5 Comparison of the major pollen taxa percentages between annual airborne pollen and surface sediment samples (for sampling locations see Fig. 1). Pollen assemblages indicate high similarities among the surface sediment samples and also high similarity to the annual airborne pollen.

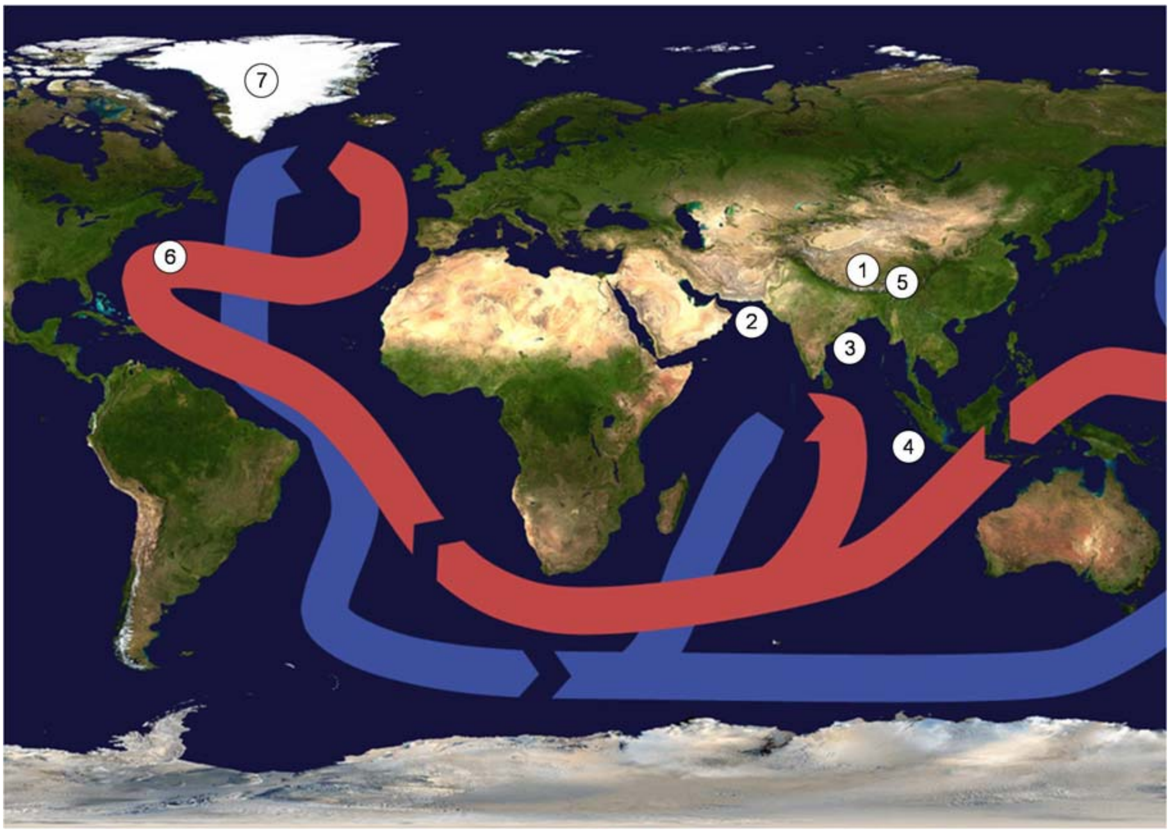

Fig. S6 Sites of records for comparison discussed in Fig. 4 and their links with the thermohaline circulation. (1) core NC08/01; (2) SO90-111KL core in the Arabian Sea; (3) SK218/1 core in the western Bengal Bay; (4) 39KL core from the eastern tropical Indian Ocean; (5) Dongge Cave of southwest China; (6) OCE326-GGC5 of the subtropical North Atlantic Ocean; (7) NGRIP core in Greenland. The map is modified from the figure "thermohaline circulation.png" from Wikimedia Commons and used under the license to copy, distribute and/or modify this document under the terms of the GNU Free Documentation License, Version 1.2 or any later version published by the Free Software Foundation (with no Invariant Sections, no Front-Cover Texts, and no Back-Cover Texts) (http://commons.wikimedia.org/wiki/File:Thermohaline_circulation.png).
